# Uterine Polypi.—II

**Published:** 1908-03-28

**Authors:** 


### GYNÆCOLOGY.

### UTERINE POLYPI.—II.

We have now to consider the fibroid polypi of the uterus, which have always the structure of a fibro myoma—that is to say, these are definitely encapsuled tumours composed of interlacing bundles of unstriated muscle cells, held together by a conspicuous stroma of fibrous tissue. The tumours are intimately connected with small arteries, and it is commonly believed that they arise by proliferation of the coats of arterioles. A pure fibroma of the uterus is practically unknown, muscle tissue being always present. The majority begin in the body of the uterus; cervical fibroids are much less common. The manner in which they become polypoid is interesting; the process is one of extrusion from the muscular wall, and nearly always inside the original capsule of the tumour. This process is common to many fibroids, and is brought about partly by growth and partly by uterine contractions. The direction in which a fibroid grows depends upon the thickness of muscle around it. When nearer the uterine cavity naturally a fibroid tends to grow into the cavity and become submucous. As soon as this happens the tumour becomes virtually a foreign body, and the uterus attempts to expel it. Hence, by muscular contraction the mass is forced down, opens up the cervical canal, and eventually appears in the vagina as a polypus. As a necessary result of this the tissues still connecting the tumour to the uterine wall become stretched and drawn out into a pedicle, carrying the blood-vessels of the growth. During this process of expulsion the uterine wall occasionally becomes inverted, but this is more likely to occur with sarcomatous growths than fibroids; because a sarcoma invades the wall, destroys the muscle at the base, and so renders it incapable of contraction.

This process of gradual extrusion of a fibroid can sometimes be surmised from the symptoms complained of by the patient. Whilst a tumour is still interstitial or submucous, simple menorrhagia is the rule, but whilst extrusion is taking place intermenstrual bleeding often occurs, or the hæmorrhage becomes continuous. The reason for this is that partial strangulation of the tumour leads to venous congestion and consequent bleeding. The onset of continuous bleeding, therefore, in a patient who is know to have a small fibroid often means that it is becoming polypoid.

On account of the great vascularity of these tumours and their exposure through the dilated cervix they are very likely to become infected, and if strangulated even partial gangrene may result. Thus the sloughing ulcers sometimes seen on fibroid polypi are set up, especially at the point of the growth exposed to the vagina. This ulcerative process may quite perforate the capsule in some cases, and the tumour may be eventually cast off altogether from the uterus. Such a termination has been known to occur after labour also, no doubt the result of some injury to the capsule from pressure.

The treatment of these polypi may be undertaken at two periods, either when they are still intra-uterine or when they are vaginal and have elongated pedicles. In the first case, as a rule, the tumour can be felt through the partially dilated cervix, and it may be advisable to remove it by enucleation through the cervix. Before doing this it is wise to ascertain, if possible, whether the tumour is solitary, for it may be that the one felt is only one of several tumours. In this case it is better, if symptoms demand it, to remove the uterus rather than the obvious tumour. If enucleation is decided upon the cervix must be dilated, or split in the middle line after separating the bladder from the uterus. The capsule is then incised and separated with the fingers from the tumour, which is held firmly gripped in a tenaculum. If the tumour is large it must be removed by "morcellement"—*i.e.* cutting out piece by piece with scissors inside the capsule. After all has been removed the capsule must be trimmed and the uterus plugged with gauze. The strictest antiseptic precautions, it need hardly be said, must be adopted in this operation, as serious infections have occurred with fatal results from them. When the tumour is quite vaginal all that is necessary is to divide the pedicle with scissors—no bleeding occurs as a rule, because the pedicle is so stretched, and retracts at once into the uterus. The écraseur was formerly employed for this purpose, but is quite unnecessary. If the tumour is so large that the pedicle cannot be reached, "morcellement" must again be made use of, successive portions being cut away until the pedicle can be felt and cut through in the ordinary manner.

Sarcomatous polypi of the uterus are occasionally seen, but are always difficult of diagnosis. As a rule such tumours are removed through the vagina, and their true nature is not realised until a microscopic examination is made. Needless to add, the uterus should always be removed at once when the true nature of the growth has been determined.

